# A successful antimicrobial regime for *Chromobacterium violaceum* induced bacteremia

**DOI:** 10.1186/1471-2334-13-4

**Published:** 2013-01-04

**Authors:** James I Campbell, Nguyen Phu Huong Lan, Phan Tu Qui, Le Thi Dung, Jeremy J Farrar, Stephen Baker

**Affiliations:** 1 The Hospital for Tropical Diseases, Wellcome Trust Major Overseas Programme, Oxford University Clinical Research Unit, Ho Chi Minh City, Vietnam; 2 The Hospital for Tropical Diseases, Ho Chi Minh City, Vietnam; 3 The London School of Hygiene and Tropical Medicine, London, UK

## Abstract

**Background:**

*Chromobacterium violaceum* is a proteobacterium found in soil and water in tropical regions. The organism rarely causes infection in humans, yet can cause a severe systemic infection by entering the bloodstream via an open wound.

**Case presentation:**

We recently identified a case of severe bacteremia caused by *Chromobacterium violaceum* at the Hospital for Tropical Diseases (HTD) in Ho Chi Minh City, Vietnam. Here, we describe how rapid microbiological identification and a combination of antimicrobials was used to successfully treat this life threatening infection in a four-year-old child.

**Conclusions:**

This case shows the need for rapid diagnosis when there is the suspicion of a puncture wound contaminated with water and soil in tropical regions. We suggest that the aggressive antimicrobial combination used here is considered when this infection is suspected.

## Background

*Chromobacterium violaceum* is a Gram-negative facultatively anaerobic proteobacterium that can be isolated from water and soil in tropical and sub-tropical regions [1]. The organism rarely infects humans; yet, occasionally, the organism can establish a severe systemic infection by entering the bloodstream via an open wound. There has been a recent surge in interest in human *Chromobacterium violaceum* infections in South East Asia, potentially as a consequence of increased reporting and awareness [2]. In 2008 we documented the first ever case in Ho Chi Minh City [3]. Since this primary case, three more infections have been observed in the city hospitals. Due to the rapid progression of human *Chromobacterium violaceum* infections, a systemic infection with this bacterium is typically fatal and no efficacious treatment regimes have ever been described. Here we report a case of *Chromobacterium violaceum* in Ho Chi Minh City that was successfully treated with a combination of antimicrobials.

## Case presentation

A four-year-old HIV negative male presented at the Hospital for Tropical Diseases in Ho Chi Minh City with a puncture wound on his right ankle. He was admitted and had a three-day history of fever, fatigue, vomiting and anorexia. He had previously been diagnosed with pulmonary Tuberculosis when three years old and had previously been taking a combination of rifampicin, 4-aminosalicylic acid and ethambutol for eight months. He had no other underlying diseases. On admission he had a pulse rate of 180 beats/minute, low blood pressure, a respiration rate of 57 breaths/minute, crackling chest sounds and pale sclera. The cervical lymph nodes were swollen, measuring 2 cm in diameter. He had displayed evidence of hepatomegaly, but was not jaundiced and had two small blisters on the abdomen.

The initial clinical diagnosis was acute sepsis. An antimicrobial regime of 200 mg/day of amikacin, 150 mg/day of vancomycin and 1.25 g/day of ceftriaxone was initiated immediately. On admission, the hematology results showed a normal white blood cell count with low hemoglobin (Table 1). The blood chemistry was also unremarkable (Table 2), apart from a C reactive protein concentration of 312 mg/l (normal range 0–5 mg/l), indicating severe sepsis. Radiography showed new pulmonary infiltrations, yet this was deemed not to be consistent with a progression of tuberculosis, a sputum smear was negative and further smears from a stomach aspirate and bronchial fluids were also negative. A blood sample was taken and inoculated into a Peds Plus/F BACTEC bottle and incubated at 37°C in an automated BACTEC 9240 machine (Becton Dickinson, United Kingdom). After 24 hours a positive result was recorded, and a Gram-negative bacilli was identified. Subcultures were performed on blood agar and nutrient agar plates and incubated aerobically at 35°C. After overnight incubation the agar plates demonstrated numerous small colonies with a dark violet metallic pigmentation. This pigmentation is unique to *Chromobacterium violaceum,* differentiating the organism from other tropical, soil dwelling organisms, and is due to the production of a chemical called violacein [4]. Identification of *Chromobacterium violaceum* was confirmed by a positive mannitol test and API 20NE [3]. The bacterial isolate was tested for susceptibility to a range of antimicrobials (cefapine, ciprofloxacin, amikacin, ofloxacin, imipenem, ceftriaxone, ceftazidime and piperacillin/tazobactam) on Mueller-Hinton agar, and interpreted according to the CLSI guidelines for non-Enterobaceriaceae Gram-negatives [5]. The phenomenon of intrinsic antimicrobial resistance in *Chromobacterium violaceum* is well described [6]. However, this isolate did not exhibit comprehensive resistant to any of the tested antimicrobials. On day three the antimicrobial therapy was changed to 330 mg/8 hours of meropenem, 150 mg/8 hours of ciprofloxacin and 150 mg/6 hours of vancomycin. This antimicrobial regime was continued for 23 days until the patient was afebrile and had no symptoms synonymous with bacteremia, additional blood cultures were not performed. The child made a complete recovery without complications.

**Table 1:** **Hematology results over *Chromobacterium violaceum *infection**

**Days**^**a**^	**WBC (K/μL)**	**% Neutrophils**	**Haemoglobin (g/dL)**	**Platelets (K/μl)**	**C-reactive protein (mg/l)**
1	8.96	77.8	11	154	312
2	9.26	83.4	14	61	298
3	8.36	84	12.8	40	241
5	4.14	57.8	11.6	50	107
6	7.85	55.6	11.4	81	93
10	14.7	62.7	9.6	392	67
12	12.85	76.9	7.4	590	51
17	14.93	69.5	11.9	635	48
18	12.37	56.7	12.2	527	16

Normal ranges, WBC; 4.3-10.8 K/μL, Neutrophils; 45 – 74%, Haemoglobin; 14 – 18 g/dL, Platelets; 150 – 350 K/μl, CRP; 0 – 5 mg/l.

^a^ Days post admission.

**Table 2:** **Blood chemistry results of a *Chromobacterium violaceum *infection on admission**

**Chemical test (normal range)**	**Result**
Sodium (135–145 mmol/l)	130 mmol/l
Potassium (3.5-5.0 mmol/l)	3.15 mmol/l
Chlorine (98–106 mmol/l)	84.8 mmol/l
Calcium (2.15-2.6 mmol/l)	2.09 mmol/l
Creatinine (53–130 μmol/l)	103 μmol/l
SGPT (0–40 Ul/l)	22 UI/l
GGT (7–50 UI/l)	24 UI/l
Lactate IV (0.6-2.4 mmol/l)	5.28 mmol/l

## Conclusions

The first reported human infection with *Chromobacterium violaceum* was in Malaysia in 1927, and less than 100 cases have been described since [7-10]. This case in Ho Chi Minh City shows the need for rapid diagnosis when there is the suspicion of a puncture wound contaminated with water and soil in tropical regions. A high C reactive protein, despite having a low specificity, was used as an indicator of severe sepsis. The subsequent early blood culture for isolation, identification and antimicrobial susceptibility were used to diagnose this infection and are essential for initiating early antimicrobial therapy. Although this type of infection is rare it should be factored into the differential diagnosis with *Burkholderia spp., Aeromonas spp. and Pseudomonas spp.* in tropical and sub-tropical regions, as a substantial delay in treatment can lead to rapid decline and death. We hope that this communication will continue to raise the awareness of this potentially fatal infection and we suggest that the aggressive antimicrobial combination used here is considered when this infection is suspected.

## Consent

Written informed consent was obtained from the patient's parent for publication of this Case report and any accompanying images. A copy of the written consent is available for review by the Editor of this journal.

## Competing interests

The authors wish to declare that they have no competing interests.

## Authors’ contributions

Conceived the study; JIC, JJF, SB, Performed microbiological culturing and identification; JIC, NPHL, LTD, Clinical treatment and antimicrobial therapy; PTQ, NPHL, Drafted manuscript; JIC, SB. All authors read and approved the final manuscript.

## Pre-publication history

The pre-publication history for this paper can be accessed here:

http://www.biomedcentral.com/1471-2334/13/4/prepub
